# A case of mosaic Goltz syndrome in a male baby: Where persistence pays off

**DOI:** 10.1016/j.jdcr.2026.06.034

**Published:** 2026-06-20

**Authors:** Yasmine Gillespie, Bliss Colao, Megan Boothe, Aaliya Ahmad, Kiran Motaparthi, Michael Joseph Lavery

**Affiliations:** a College of Medicine, University of Florida, Gainesville, Florida; b Department of Dermatology, University of Florida College of Medicine, Gainesville, Florida; c Division of Genetics and Metabolism, University of Florida, Gainesville, Florida

**Keywords:** focal dermal hypoplasia, Goltz syndrome, lines of Blaschko, mosaicism, X-linked dominant disorders

## Introduction

Goltz syndrome, also known as focal dermal hypoplasia or Goltz-Gorlin syndrome, is a rare genetic disorder characterized by abnormalities involving the integumentary, skeletal, gastrointestinal, genitourinary, cardiovascular, ophthalmic, dental, and nervous systems.^1^^,^^2^

This is an X-linked dominant disorder caused by loss-of-function variants in *PORCN*, which encodes porcupine O-acyltransferase, an enzyme essential for wingless-related integration site proteins during fetal development.^3^ Approximately 300 cases have been reported, mostly being female as the condition is typically male-lethal in utero. Rare male cases of Goltz are due to postzygotic mutations resulting in somatic mosaicism for pathogenic *PORCN* variants have been reported.^4^ Diagnosing Goltz syndrome in males can be challenging, as the rarity of the condition means there is not a well-described phenotype. It is often difficult to confirm the presence of a pathogenic *PORCN* variant due to somatic mosaicism. Here, we expand the phenotype of males with Goltz syndrome by presenting a previously unreported case of Goltz syndrome in a male patient.

## Case presentation

Our patient was born at 39 weeks to a 33-year-old woman with limited prenatal care. The parents reported 2 other healthy children. The patient was apneic upon delivery, intubated and admitted to the neonatal intensive care unit. He had multiple congenital abnormalities, including a left congenital diaphragmatic hernia (Fig 1).

**Fig 1:**
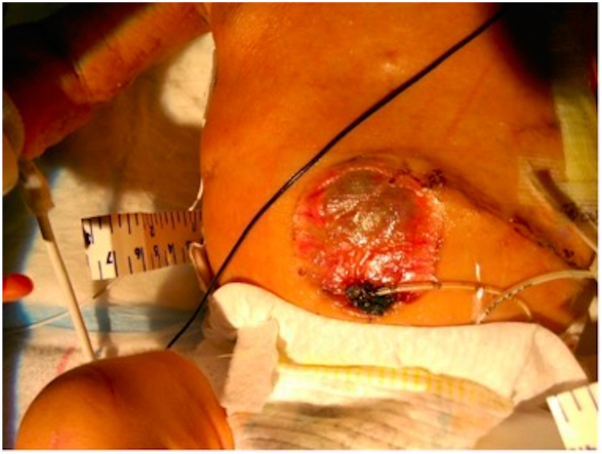
Clinical presentation of patient’s omphalocele.

Examination on day 1 of life showed nodular fat herniations, linear atrophic hyperpigmented streaks along the lines of Blaschko on the arms, inguinal folds, flank, thighs, and groin (Fig 2, *A* and *B*), small omphalocele, and bilateral syndactyl polydactyl with lobster claw deformity (Fig 3).

**Fig 2:**
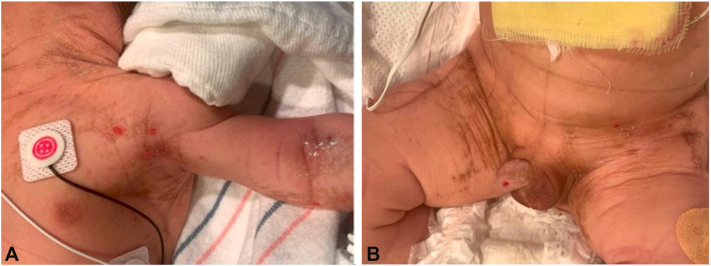
**(A)** Hyperpigmentation along Blaschko’s lines on patient’s chest and left upper extremity. **(B)** Hyperpigmentation along Blaschko’s lines on patient’s abdomen, groin, and lower extremities.

**Fig 3:**
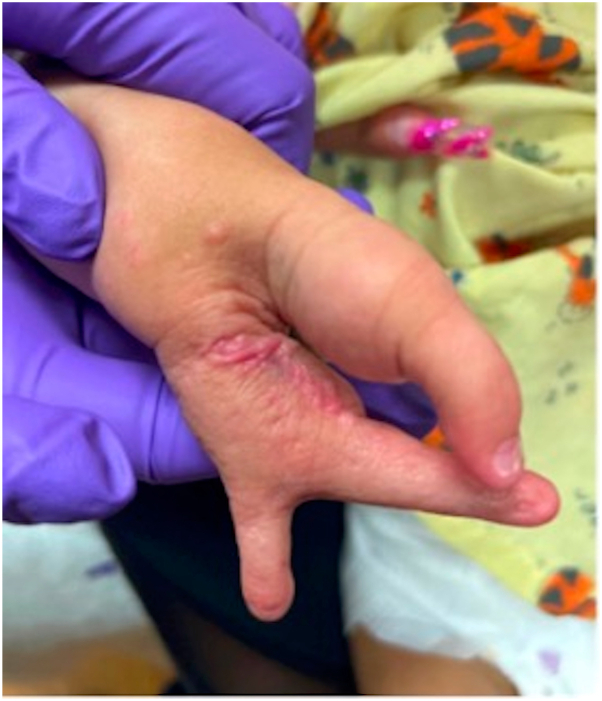
Photograph of syndactyl polydactyl with lobster claw deformity.

Differential diagnosis at birth included Goltz syndrome, Pentalogy of Cantrell, Adams-Oliver syndrome, Pallister-Killian syndrome (mosaic tetrasomy 12p), and Fryns syndrome. The diaphragmatic hernia and omphalocele were surgically repaired. Chromosome microarray and whole exome sequencing on blood was negative. Follow-up *PORCN* gene analysis and chromosome microarray on buccal sample were also negative.

The patient then presented at 1 year with a verruciform papule at the left oral commissure (Fig 4), consistent with the clinical diagnosis of an oral papilloma. He was referred to plastic surgery for excision of the lesion and given the high index of suspicion for Goltz syndrome, genetic analysis was performed on the papilloma.

**Fig 4:**
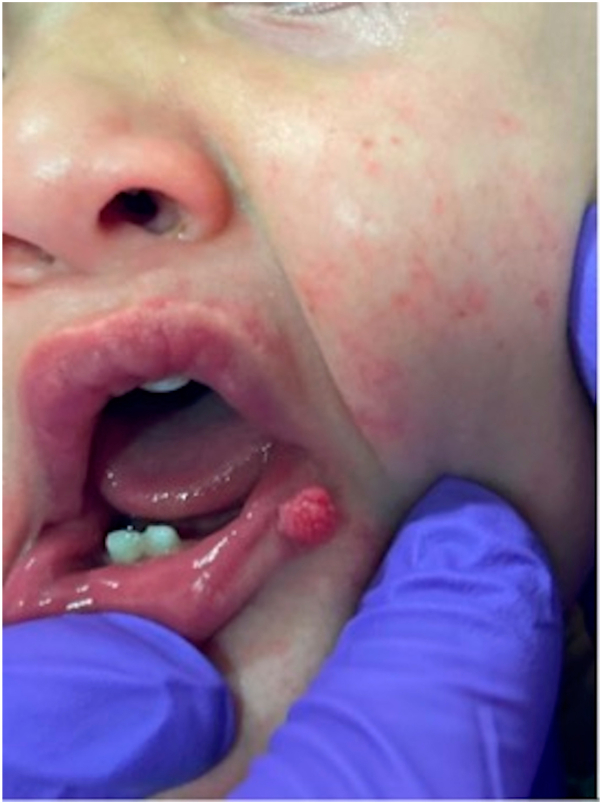
Clinical presentation of oral papilloma on patient’s left oral commissure.

This deletion was classified as pathogenic, as haploinsufficiency of *PORCN* is a known mechanism of disease and similar Xp11.23 deletions have been reported in other individuals with Goltz syndrome.^5^^,^^3^ A level of mosaicism in the tissue sample could not be precisely determined by the testing methodology. The patient has regular follow-up with dermatology, cardiology, gastroenterology, pulmonology, ophthalmology, dentistry, and genetics.

## Discussion

This case highlights the diagnostic complexity of Goltz syndrome, particularly in males in whom mosaicism may obscure early genetic confirmation. Goltz syndrome can present with various cutaneous and systemic manifestations. Characteristic cutaneous findings include patchy skin aplasia, nodular fat herniation, hyperpigmentation or hypopigmentation along lines of Blaschko, telangiectasias, dysplastic nails, lipomatous changes, verrucous papillomas, sparse hair, and patchy alopecia.^6^^,^^5^ Cutaneous features can vary due to X-inactivation patterns in affected females and somatic mosaicism in affected males.^6^

Other abnormalities seen in Goltz syndrome can include missing, hypoplastic, or abnormal teeth; and anophthalmia, microphthalmia, chorioretinal coloboma, lacrimal duct abnormalities, and corneal clouding.^6^^,^^5^ Craniofacial abnormalities, such as facial asymmetry, pointed chin, broad nasal tip, narrow nasal bridge, cleft lip and palate, notched alae nasi, and small underfolded pinnae, may be observed.^6^ Abdominal wall defects have also been seen, mainly as a diaphragmatic hernia.^5^ Finally, while some patients have normal cognitive development, some may exhibit impairment and/or behavioral issues.^5^

A distinct feature of Goltz syndrome is distribution of cutaneous findings along the lines of Blaschko, which represent migration of epidermal cells during fetal development. Blaschko lines are separate from dermatomes. This pattern is a common hallmark among several X-linked dominant conditions including Incontinentia pigmenti, congenital hemidysplasia with ichthyosiform erythroderma and limb defects syndrome, microphthalmia with linear skin defects syndrome, Oral-Facial-Digital Syndrome Type 1, and X-linked dominant chondrodysplasia punctata.^7^^,^^8^ The patient’s presentation is most consistent with postzygotic somatic mosaicism.

One condition that shares some overlapping features with Goltz syndrome is Pentalogy of Cantrell. This condition, which lacks a clear genetic etiology, is defined by 5 congenital midline defects, including omphalocele, diaphragmatic hernia, sternal cleft, intracardiac defect, and ectopia cordis.^9^ In our case, Pentalogy of Cantrell was on the differential diagnosis in the neonatal intensive care unit.^9^ Our patient had an omphalocele and diaphragmatic hernia but lacked other manifestations.

This case underscores the challenges of diagnosing rare genetic disorders like Goltz syndrome. While Goltz is more common in females, rare cases occur in males due to somatic mosaicism. Mosaic variants may be challenging to diagnose, as the mutation is only present in a subset of cells. In our case, buccal and serum samples were negative for the pathogenic *PORCN* variant (germline), while the affected tissue was positive (postzygotic somatic mutation). Clinicians should maintain a high suspicion for X-linked dominant conditions in male patients. Other rare etiologies of Goltz syndrome in male patients include Klinefelter’s syndrome (XXY) and hypomorphic variants resulting in decreased, but not absent, *PORCN* function. Prompt investigation is crucial when clinical suspicion is high, necessitating multidisciplinary collaboration.

Following the diagnosis of Goltz syndrome, consideration should also be given to future reproductive risk. In this case, the mutation is postzygotic somatic mosaicism, which significantly reduces the risk for parents to have an affected child. However, affected males may have up to a 100% risk of transmitting the mutation to daughters depending on germline involvement, while the risk to sons remains extremely low due to paternal X-chromosome inheritance.

Multidisciplinary collaboration is essential for navigating the complexities of such cases, facilitating comprehensive evaluation and management strategies tailored to individual patient needs. In conclusion, our case highlights the importance of heightened clinical suspicion, meticulous examination, and timely genetic testing in unraveling the intricate phenotypic manifestations of rare genetic disorders like Goltz syndrome.

## Conflicts of interest

None disclosed.
